# An Account of the Good Effects of Mercury in Two Cases of Impeded Deglutition; to Which Is Added an Instance of the Relief Obtained from the Same Remedy in a Spasmodic Affection of the Neck of the Bladder

**Published:** 1789

**Authors:** Samuel Patten

**Affiliations:** Surgeon in London


III. *An Account of the good Effects of Mercury in two Caſes of impeded Deglutition; to which is added an Inſtance of the Relief obtained from the ſame Remedy in a ſpaſmodic Affection of the Neck of the Bladder. By Mr.* Samuel Patten, *Surgeon in London. Communicated in a Letter to* J. H. Sequiera, *M. D. Phyſician in London; and by him to Dr.* Simmons.

*To Dr.* SEQUIERA.

SIR,

THE good effects of the mercurial courſe recommended by you in the caſe of Mrs.

 Merritt,

Merritt, who laboured under a difficulty of ſwallowing, having ſince induced me to have recourſe to a ſimilar mode of treatment in another inſtance of the ſame kind, which likewiſe terminated ſucceſsfully, I take the liberty of tranſmitting a ſhort account of both the caſes to you, with a requeſt that you will diſpoſe of them as you may think proper.

They may, perhaps, ſerve as a ſupplement to your paper on this ſubject in the ſixth volume of the Medical Obſervations and Inquiries; and will probably be deemed the more deſerving of attention, as the caſes of this ſort, upon record, ſince the publication of the late Dr. Munckley's hiſtory and cure of a dangerous affection of the œſophagus *, are but few in number.

I have the honour to be, SIR,

&c.

### CASE I.

In the month of Auguſt, 1782, I was deſired to viſit Mrs. Anne Merritt, of Ratcliffe Croſs, a married woman, twenty-two years of age, who, after taking cold, had been ſeized

* See Medical Tranſactions, Vol. I. page 165.

with with a dimneſs of ſight, and a difficulty of ſwallowing.

Her ſight, which, till the commencement of her preſent complaint, had been perfect, was at the time I firſt ſaw her ſo much impaired, that ſhe could not ſee to thread a very large needle; and her deglutition was ſo much impeded, that it was not without great trouble ſhe was able to ſwallow liquids; but ſolid food paſſed down with ſomewhat leſs difficulty.

Theſe complaints had begun and increaſed for ſome days before I was called to her aſſiſtance. I immediately applied leeches to the temples, and bliſters behind the ears; but neither theſe nor the uſe of laxative and volatile medicines, to both of which I had recourſe, with the occaſional uſe of antimonial wine, produced any alleviation of the ſymptoms.

Her eyes were perfectly clear, but the pupils ſeemed to be ſomewhat more dilated than uſual, though ſufficiently acted upon by the light.

No ſwelling, or appearance of inflammation, could be diſcovered in the tonſils or fauces; and the only thing obſervable about the throat was a fulneſs, externally, on one ſide of the neck: this part was frequently rubbed with volatile liniment, but without any good effect.

As

As the different remedies I tried had proved ineffectual, and ſhe experienced great difficulty in getting down the medicines, ſhe determined to lay them aſide; and I ſuſpended my viſits for about a week, when I again called on her. She was then ſo much weakened and emaciated, from not being able to get down nouriſhment, that her ſituation appeared to be very alarming.

At this period of the caſe you was conſulted; and it was agreed to try the effects of mercury in a quantity ſufficient to excite and, if neceſſary, keep up a gentle ptyaliſm. The *ung. mercur. fort.* was accordingly employed for this purpoſe; a pill containing a grain of calomel was at the ſame time given every night; and the *ceratum mercur.* ſpread upon linen was conſtantly worn by the patient on the fore part of her neck.

This method was purſued during ten days, when her mouth began to be affected, and the ſymptoms ſubſided, both the ſight and power of ſwallowing having been gradually reſtored; ſo that at the end of three weeks the patient found herſelf quite well.

CASE

### CASE II.

The ſecond inſtance, in which I had occaſion to obſerve the good effects of a ſimilar mode of treatment, occurred in the month of December, 1782. The patient was a young lady at Stepney, who, after taking cold, began to complain of a ſpaſmodic affection of the œſophagus, which made it very difficult for her to ſwallow liquids; though any thing ſolid, by acting againſt the ſpaſm of the œſophagus, could be forced down with more eaſe.

No appearance of inflammation was preſent; and the ſucceſs that had attended the uſe of mercury in Mrs. Merritt's caſe determined me to have recourſe to it in this. I accordingly gave her ſmall doſes of calomel, which, in the ſpace of about eight days, occaſioned a ſoreneſs of the mouth, and the complaint then gave way. In this caſe no ptyaliſm took place.

I have ſince that time experienced the efficacy of mercurial remedies in ſpaſmodic affections of the neck of the bladder; particularly in the caſe of a female patient, in which no marks of inflammation ever appeared, and in which there was not any reaſon to ſuſpect that a ſtone was preſent. An interval of more than two two years occurred between the two attacks; and in each of the paroxyfms the pain and efforts to void the urine were extremely violent. In the firft attack this patient took laxative medicines, and large dofes of opium, without any confiderable alleviation of the complaint. Calomel was then adminiftered, in fmall dofes, and a gentle fpitting was no fooner, by this means, excited, than the diforder gave way. In the fecond attack I immediately had recourfe to the fame remedy, and experienced from it the fame good effects as before.

*Ratcliffe Crofs,*
July 29, 1789.

